# Oxidation and Ablation Behavior of Particle-Filled SiCN Precursor Coatings for Thin-Film Sensors

**DOI:** 10.3390/polym15153319

**Published:** 2023-08-07

**Authors:** Lanlan Li, Yingping He, Lida Xu, Chenhe Shao, Gonghan He, Daoheng Sun, Zhenyin Hai

**Affiliations:** 1 Department of Mechanical and Electrical Engineering, School of Aerospace Engineering, Xiamen University, Xiamen 361102, China; lilanlan1006@163.com (L.L.); hyp19980504@163.com (Y.H.); superldxu@gmail.com (L.X.); chenhe2019@stu.xmu.edu.cn (C.S.); hgh@xmu.edu.cn (G.H.); 2 Fujian Micro/Nano Manufacturing Engineering Technology Research Center, Xiamen University, Xiamen 361102, China

**Keywords:** polymer-derived ceramics, thin-film coatings, oxidation, ablation

## Abstract

Polymer-derived ceramic (PDC) thin-film sensors have a very high potential for extreme environments. However, the erosion caused by high-temperature airflow at the hot-end poses a significant challenge to the stability of PDC thin-film sensors. Here, we fabricate a thin-film coating by PDC/TiB_2_/B composite ceramic material, which can be used to enhance the oxidation resistance and ablation resistance of the sensors. Due to the formation of a dense oxide layer on the surface of the thin-film coating in a high-temperature air environment, it effectively prevents the ingress of oxygen as a pivotal barrier. The coating exhibits an exceptionally thin oxide layer thickness of merely 8 μm, while its oxidation resistance was rigorously assessed under air exposure at 800 °C, proving its enduring protection for a minimum duration of 10 h. Additionally, during ablation testing using a flame gun that can generate temperatures of up to 1000 °C, the linear ablation rate of thin-film coating is merely 1.04 μm/min. Our analysis reveals that the volatilization of B_2_O_3_ occurs while new SiO_2_ is formed on the thin-film coating surface. This phenomenon leads to the absorption of heat, thereby enhancing the ablative resistance performance of the thin-film sensor. The results indicate that the thin-film sensor exhibits exceptional resistance to oxidation and ablation when protected by the coating, which has great potential for aerospace applications.

## 1. Introduction

The growing demand for monitoring operations in harsh environments has stimulated the advancement of high-temperature sensors. High-temperature thin-film sensors offer significant potential for integration into high-temperature components owing to their advantageous attributes, including micrometer-scale thickness, negligible mass, non-intrusive nature, and minimal interference with surface airflow and component vibration modes [1,2,3]. Compared with metal materials, the precursors of PDC are generally liquid polymers, and the liquid precursors forming-curing-crosslinking-pyrolysis to form a ceramic structure. Its working ability in extreme environments, such as oxidation resistance and thermal shock resistance, is significantly better than alloys. Polymer-derived ceramics (PDCs), due to their excellent resistance to oxidation and high temperature properties [4], are widely used in high-temperature bulk [5,6,7] and thin-film sensors. PDC thin-film sensors [8] can be fabricated by an in situ direct writing method, which has a great advantage in the high-temperature sensor field.

Currently, a significant amount of research is focused on exploring the performance of PDC thin-film sensors, including strain gauges [8,9], temperature sensors [10,11,12], and heat flux sensors [13]. Cui [14] developed a temperature-sensitive sensor using a SiCN film that was less than 100 μm thickness, with a maximum measurement temperature of 800 °C. Wu et al. [9] fabricated TiB_2_/SiCN thin-film strain gages, which can work close to 800 °C. While most of these fabricated films exhibit stable operation at temperatures ranging from 25 °C to 800 °C, their long-term stability without peeling or oxidation in real high-temperature operating environments is crucial. Therefore, it is crucial to develop an anti-oxidation and anti-ablation thin-film coating for thin-film sensors. Cui et al. [15] successfully prepared a PDC antioxidant coating that can be applied to thin-film sensors up to 800 °C. Xu et al. [16] developed a double-layer high-temperature antioxidant PDC composite film by direct writing. Wu et al. [17] improved the high-temperature stability of SiCN thin-film resistive grids by fabricating Al_2_O_3_/SiCN composite films. These studies, however, did not investigate the oxidation resistance of the films in depth. Since the PDC composite film is used as an antioxidant layer, it also needs to have resistance to the effect of high-temperature airflow in harsh environments [18,19]. However, their investigation only made a preliminary investigation of the antioxidant properties of the film without studying the ablation resistance of the film. 

Although there have been a large number of studies on the ablation resistance of coatings [20,21,22,23,24], Yanjiang et al. [20] prepared TZSS coatings encapsulating C/C substrates with a size of 10 mm × 5 mm × 10 mm, which can be ablated at 4.2 MW/m² for 40 s. Jing’an Kong et al. [21] prepared TaC coatings and performed cyclic oxyacetylene flame tests with a coating thickness of 600 μm. However, these coatings were prepared on C/C substrates with thickened thicknesses. Unlike the bulk coating, the thin-film coating is fabricated in situ on top of the alumina substrate or on top of the thin-film thermistor with a coating thickness in the micron range. The ablation resistance studies of coatings have predominantly concentrated on C/C composites. Unlike these coatings, thin-film coatings are fabricated in situ on substrates or thin-film sensors with specimen thicknesses at the micron level [15,17]. Meanwhile, the investigation into the ablation resistance of thin-film coatings has been limited. Nonetheless, it is crucial to carry out comprehensive studies on the coupling behavior between film oxidation and ablation for practical applications.

In this work, through our analysis of the oxidation and ablation behavior of PDC TiB_2_/B composites, we observed that the formation of SiO_2_-B_2_O_3_ on the coating surface is vital for improving the oxidation and ablation resistance of the thin-film sensors. The SiO_2_-B_2_O_3_ oxide layer formed at high temperature is 7~8 μm thick and its well prevented the entry of oxygen. Furthermore, we characterized the ablation process of the film using a butane flame at 1000 °C with a linear ablation rate of 1.04 μm/min. During the ablation process, the B_2_O_3_ formed on its surface volatilized, and the SiCN thermally decomposed to generate new SiO_2_, which absorbed the energy of ablation and played a key role in protecting the sensitive layer of the thin-film sensor. And it was observed that the thin-film sensor maintained a resistance change of 0.075%/min of exposure to the flame spraying test. The results demonstrate that the PDC/TiB_2_/B composite thin-film coating enhances the oxidation and ablation resistance of thin-film sensors at high temperatures, making it an option for protective coatings in demanding environments, such as aerospace applications. 

## 2. Experimental Section

### 2.1. The Fabrication of Thin-Film Coatings

In this study, PSN2 (commercially provided by the Institute of Chemistry, Chinese Academy of Sciences, Beijing, China) was used as the precursor of SiCN. TiB_2_ and B powders were used as the filler particles with an average diameter of 1 μm (Shanghai Chaowei Nano Technology Co., Shanghai, China). And there was an alumina base (15 mm × 15 mm × 1 mm) that was used for oxidation and ablation performance tests. 

**Figure 1:**
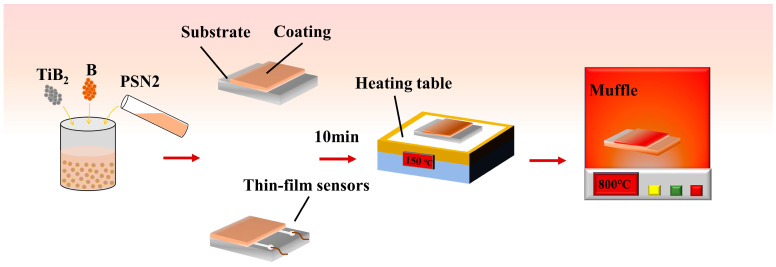
Fabrication process of the coating.

As shown in Figure 1, the preparation process of the coating is described. First, PSN2, B and TiB_2_ are mixed according to the mass ratio of 1:0.8:1 to prepare a mixed solution. The PDC composite ink is put on the magneton mixer and stirred at room temperature for 2 h. Next, using the microscale Wie-senberg platform, the mixed ink is written directly onto the alumina substrate. After the direct writing is complete, it is placed on a heated platform and cured at 100 °C for 10 min. Finally, the film is annealed in the air at 800 °C in the muffle furnace and cooled with the furnace; it reaches the indoor temperature, and can be taken out. The thin-film coating is thus obtained. 

Currently, we are applying the prepared thin-film above the thin-film sensor. By coating the thin-film on the alumina substrate where the sensitive gate is fabricated, Please delete it. The correct statement should be: we can obtain a double-layer thin-film sensor [14]. The resistance of this sensor is characterized by cycling stability at room temperature to 800 °C [14].

### 2.2. Protection Performance Tests of the Thin-Film Coatings

The furnace was set to 800 °C to test the oxidation resistance of the coating. A thermogravimetric analyzer (TG-DSC, TGA/DSC, STA449F5) was used for the analysis of TiB_2_, B and thin-films. At 800 °C, the oxidation characteristic time was heated at a rate of 10 K/min. The following equation calculated cumulative mass change percentages (∆mass%) of the powders:

$$\Delta \mathrm{mass}\%=\frac{m_{t}-m_{0}}{m_{0}}$$

where $m_{0}$ and $m_{t}$ are the mass of the powder before and after oxidation for *t* minutes. The curves of mass change with oxidation time were given according to the above calculation formula.

**Figure 2:**
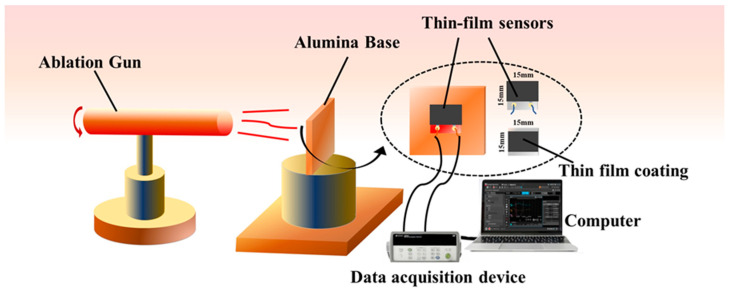
Simple system for film ablation resistance.

To assess the ablation resistance of the film, a simplified film ablation system was established (Figure 2). The system utilized a flame gun powered by a 95% butane gas stream, with a test temperature of 1000 °C. The flame gun nozzle had an inner diameter of 20 mm and was positioned 6 cm away from the sample. The butane gas flow rate was set at 0.03 L/s. A thermocouple was centrally placed on the sample to monitor the real-time temperature. The film underwent continuous ablation in the flame for durations of 4 min, 10 min, 15 min, and 30 min, and the linear ablation rate was subsequently calculated. The line ablation rate was used to evaluate the ablation resistance.

$$R_{m}=\frac{\Delta m}{t}$$

where $R_{m}$ is the line ablation rate, ∆*m* is the coating thickness change, and *t* is the ablation time.

### 2.3. Characterization

The sensors were primarily characterized using a profilometer (Dektak XT) to measure their thickness, and scanning electron microscopy with energy-dispersive spectroscopy (SEM-EDS) using a JSM-IT500A instrument from JEOL in Tokyo, Japan, to analyze their morphology and elemental composition. X-ray diffraction (XRD) analysis was performed using a Shimadzu XRD-6100 instrument. The output resistance of the thin-film sensors was measured using a Keysight 34972A data acquisition device (DAQ).

## 3. Results and Discussion

### 3.1. Phase Analysis and Microstructure of the Thin-Film Coatings

**Figure 3:**
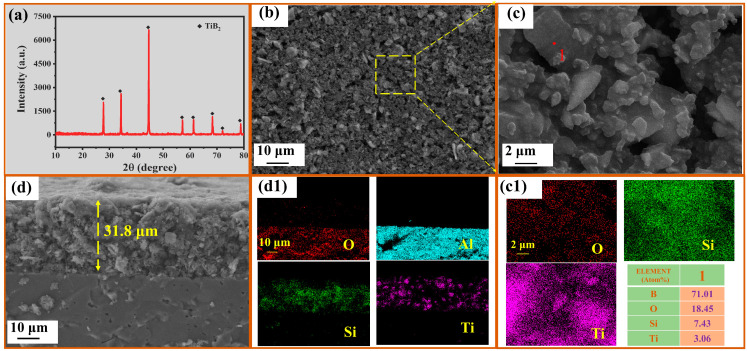
(**a**) XRD pattern of the surface; (**b**) Surface image of the prefabricated coating; (**c**) local surface of (**a**); (**c1**) EDS analysis of (**c**) and spot 1; (**d**) cross-sectional image of the coating; (**d1**) EDS analysis of (**d**).

Figure 3a shows the XRD pattern of the prefabricated coating; the TiB_2_ phases are detected. Figure 3b,c depict scanning electron microscope (SEM) images of the prefabricated coating surface, providing insight into the distribution and morphology of the raw material particles. The surface exhibits a relatively rough texture and contains noticeable particulate features. EDS analysis of spot 1 (Figure 3c) reveals the presence of titanium (Ti) and boron (B) on the surface. Furthermore, Figure 3(c1) exhibits a uniform distribution of oxygen (O) and silicon (Si) elements, indicating the effective bonding role of PSN2 during the coating fabrication process. Consequently, the surface particles primarily consist of TiB_2_ and B. Figure 3d displays a cross-sectional view of the prefabricated coating, showcasing a uniform thickness of approximately 30 μm applied to the alumina surface. The Energy Dispersive Spectrometer (EDS) analysis (Figure 3(d1)) clearly distinguishes titanium (Ti) elements and reveals relatively even distribution of silicon (Si) and aluminum (Al) elements, where Al is the element of the base (Al_2_O_3_). Notably, no instances of delamination or peeling are observed, except for a few larger TiB_2_ particles measuring 1 μm. The coating is successfully obtained at room temperature and exhibits excellent adhesion to the alumina substrate.

### 3.2. Oxidation Behavior of the Thin-Film Coatings

**Figure 4:**
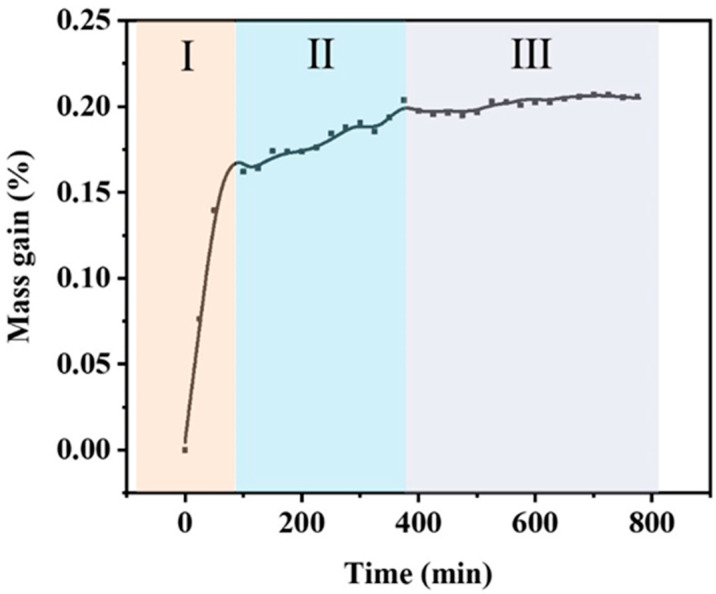
Isothermal oxidation curves of the coating.

**Figure 5:**
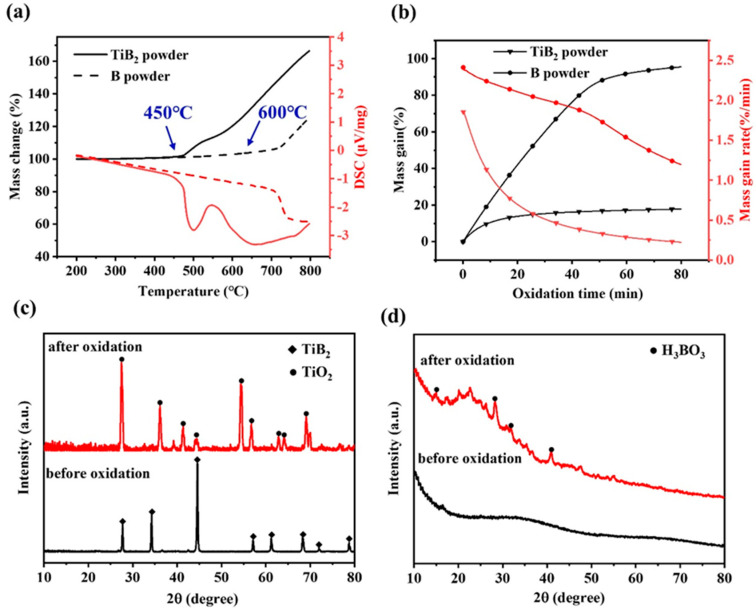
(**a**,**b**) TG−DSC and isothermal oxidation curves of TiB_2_ and B powders, respectively; (**c**,**d**) XRD patterns of the TiB_2_ and B powders after oxidation in 800 °C.

To evaluate the oxidation resistance of the coating, we obtained an isothermal curve by subjecting the coated material to a temperature of 800 °C for 12h and air flow of 50 mL/min (Figure 4). The curve exhibits three distinct stages: a rapid mass increase (Stage I), a slower mass increase (Stage II), and a plateau (Stage III). After 80 min of oxidation of the coating, its mass increased sharply, reaching 0.15 wt%. According to the TG-DSC and isothermal oxidation curves (Figure 5a,b) of B powder and TiB_2_ powder, the furnace is heated up to 800 °C with a rate of 10 °C/min, and keeping for 50 mL/min of the air flow. It can be seen that there is a large amount of oxygen diffusion in the powder, oxidizing TiB2 and B particles, producing TiO_2_ and B_2_O_3_ (Figure 5c,d). Based on the literature of the antioxidant thin-film coating, it is required to have a good coefficient of thermal expansion with the substrate and sensitive layers [25,26]. The average alumina substrate thermal expansion coefficient [27] is measured at 7.1 × 10^−6^/K^−1^. Additionally, the average thermal expansion coefficients of B and TiB_2_ are 6.4 × 10^−6^/K^−1^ and 7.0 × 10^−6^/K^−1^, respectively [28,29]. Notably, TiB_2_ is a well-established high-temperature material that has been successfully utilized in high-temperature thin-film applications [9,30]. Consequently, the combination of B/TiB_2_ offers significant advantages over using only B as the filler, as it achieves a superior thermal match with both the substrate and the sensitive layer of the thin-film sensor. This enhanced thermal match ensures improved overall performance and stability for the thin-film sensor. As oxidation time increases, mass gain enters a slow phase (II stage). The mass plateau stage is in III stage with a mass gain of 0.2 wt%. 

**Figure 6:**
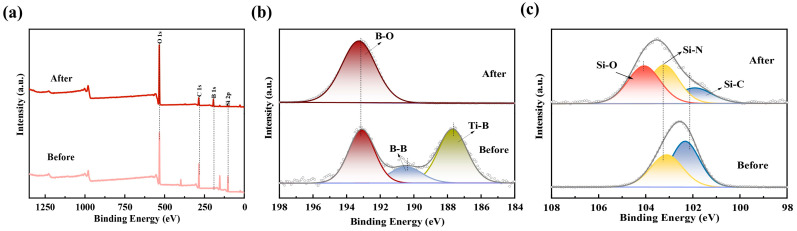
The sample surface before and after exposure to 800 °C (**a**) Full spectrum of XPS. (**b**) B 1s spectra and (**c**) Si 2p spectra before and after exposure to high temperature.

It is also found that the initial oxidation temperature of TiB_2_ powder in Figure 5a is about 450 °C [31], which is significantly lower than that of B powder at 600 °C [32]. At the same time, the oxidation trend of TiB_2_ in air is significantly greater than that of B. Therefore, the presence of B allows the coating to better form an oxide layer. At the same time, SiCN undergoes a high-temperature reaction, leading to the generation of SiO_2_, which effectively repairs any cracks caused by oxidation. And the changes of substances before and after exposure to high temperatures of the thin-film coatings are analyzed using X-ray photoelectron spectroscopy (XPS). The full spectra (Figure 6a) reveal a noticeable increase in the concentration of O and B on the sample surface following exposure to high temperatures. The Si 2p spectrum (Figure 6b) demonstrates the presence of Si-O bonds (at 104.3 eV), indicating the formation of SiO_2_. On the other hand, the analysis of the B 1s spectrum (Figure 6c) confirms the existence of solely B-O bonds after the high-temperature treatment. The possible oxidation reactions were shown as follows [33,34,35]

2SiCN + 3O_2_→2SiO_2_(l) + 2CO+N_2_(g)

2TiB_2_ + 5O_2_→2TiO_2_ + 3B_2_O_3_

4B + 3O_2_→2B_2_O_3_(l)

SiO_2_(l) + B_2_O_3_(l)→SiO_2_·B_2_O_3_(l)

**Figure 7:**
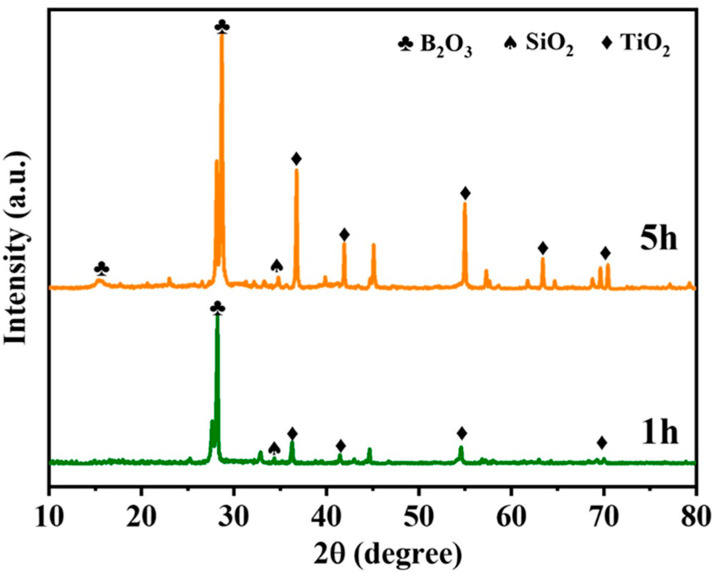
XRD patterns of the coating after oxidation for different times.

**Figure 8:**
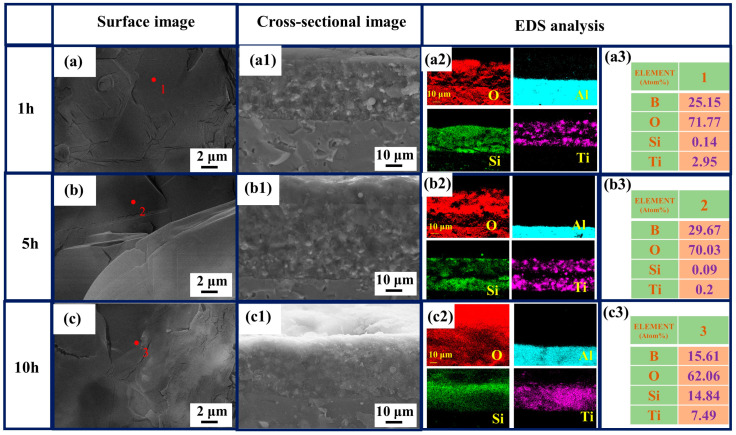
Surface and cross-sectional topography of the coating after oxidation for different times: 1 h (**a**–**a3**); 5 h (**b**–**b3**); 10 h (**c**–**c3**); element distribution map (**a2**,**b2**,**c2**) of the SEM image (**a1**,**b1**,**c1**); EDS analysis (**a3**,**b3**,**c3**) of spot 1, 2 and 3 (**a**,**b**,**c**).

The phase transformation of the coating during the oxidation process was investigated by XRD analysis for different oxidation times (Figure 7). After 1 h of oxidation, SiO_2_, B_2_O_3_ and TiO_2_ phases appeared. It showed that a glassy oxide layer was formed on the surface of the coating, and after 5 h, the peak strength of B_2_O_3_ (Figure 7) increased, indicating that the density of the antioxidant layer was also enhanced (Figure 8b,c). Figure 8 shows the evolution of surface and cross-sectional topography during oxidation of the coating. After oxidation at 800 °C for 1 h (Figure 8a), 5 h (Figure 8b), 10 h (Figure 8c), an oxide film is formed on its surface, which is dense and has no micropores. According to the specific gravity analysis of the elements of points EDS (Figure 8(a3,b3,c3)), the O content is higher, followed by the B content. Combined with the results of XRD (Figure 7), a dense B_2_O_3_· SiO_2_ glass layer forms on the surface of the coating. At the same time, the cross-sectional topography of the coating is analyzed. After 1 h of oxidation (Figure 8(a1,a2)), the O element on the outside of the coating is higher than the inside, and the distribution of Ti elements is granular, indicating that the particles inside the coating are not completely oxidized. After 10 h of oxidation (Figure 8(b1,b2)), a dense oxide layer (thickness: ~8 μm) is formed on the surface of the coating, the surface of which is covered by a thick glass layer.

### 3.3. Ablation Behavior of the Coated Samples at Different Times

**Figure 9:**
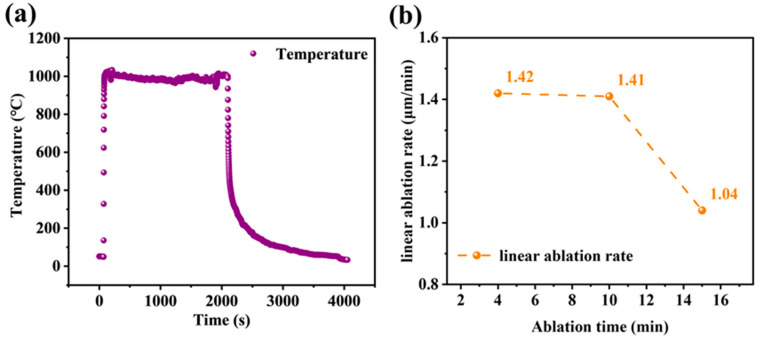
(**a**) Ablation center temperature curves; (**b**)Variations of linear ablation rates.

To evaluate the protective performance of antioxidant films against erosion in high-velocity airflow environments, a flame spray apparatus was employed to assess their ablation resistance. Figure 9a illustrates the film's ablation process at a central temperature of 1000 °C and the thickness change of the ablation pit is calculated. Figure 9b presents the linear ablation rates of the film samples at different time intervals, revealing rates of 1.42 μm/min, 1.41 μm/min, and 1.04 μm/min at 4 min, 10 min, and 15 min, respectively. These results indicate a progressive reduction in film thickness and ablation rate as the duration of ablation increases. At the same time, the line ablation rate decreases with time.

**Figure 10:**
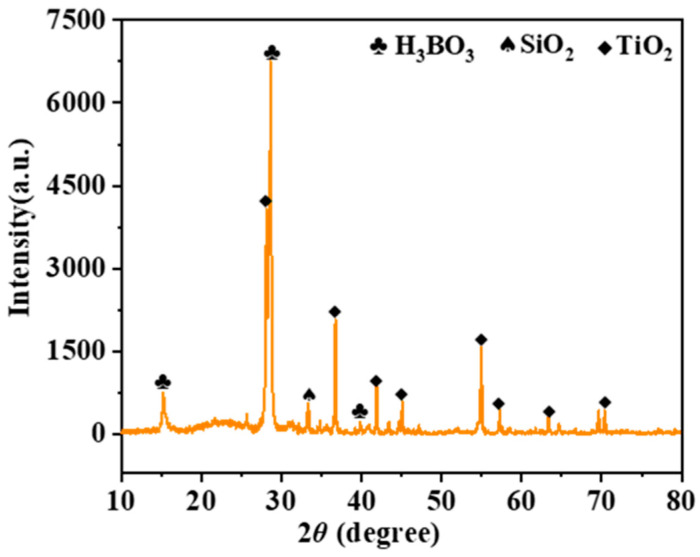
XRD patterns of the coating after ablation in flame.

**Figure 11:**
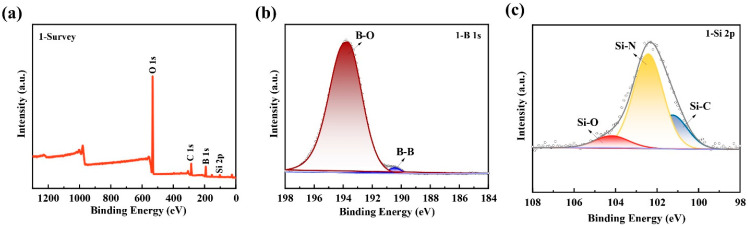
The sample surface after ablation (**a**) Full spectrum of XPS. (**b**) B 1s spectra and (**c**) Si 2p spectra before and after exposure to high temperature.

**Figure 12:**
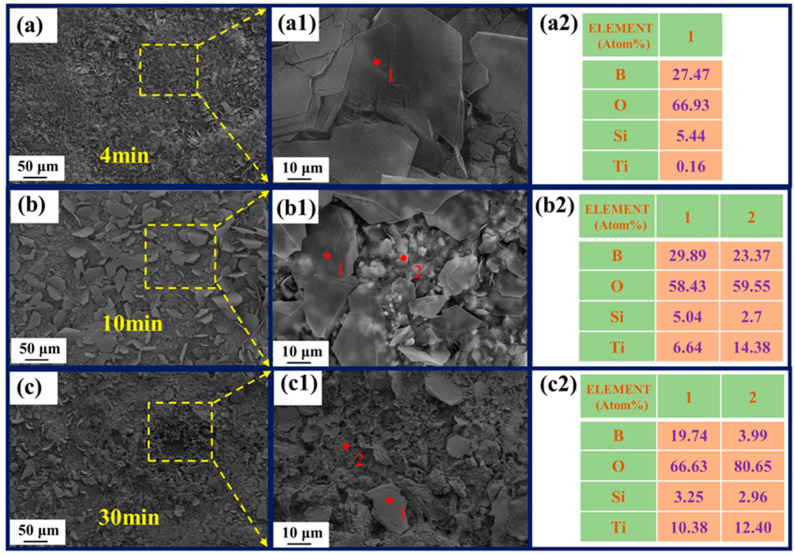
Surface SEM micrographs of different times: 4 min (**a**–**a2**); 10 min (**b**–**b2**); 30 min (**c**–**c2**); EDS analysis (**a2**,**b2**,**c2**) of spot 1, 2 (**a1**,**b1**,**c1**).

**Figure 13:**
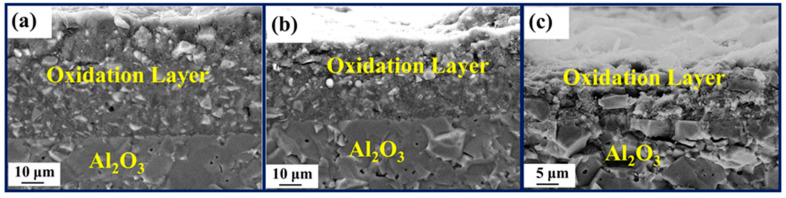
Cross-section SEM micrographs of different times: 4 min (**a**); 10 min (**b**); 30 min (**c**).

Figure 10 displays the XRD spectrum of the ablated film samples, demonstrating the presence of residual oxides primarily composed of B_2_O_3_, TiO_2_, and SiO_2_ on the surface. The oxide (B_2_O_3_ and SiO_2_) appears on the sample surface according to XPS (Figure 11), although their content is relatively less than annealing completed. The Si-O bond content was 9.43% (Figure 11c), lower than 41.66% (Figure 6c). SEM images (Figure 12(a,a1)) reveal the film's intact morphology without visible voids or cracks after 4 min of ablation. However, after 10 min of ablation, surface melting occurs, exposing TiB_2_ and TiO_2_ particles (Figure 12(b1,b2)). The low melting point of B_2_O_3_ (450 °C) leads to the volatilization of the surface oxides under the high-temperature conditions of 1000 °C. After 30 min of ablation, distinct ablation pits appear on the surface, accompanied by a decrease in the proportion of B and Si as observed through EDS analysis (Figure 12(a1,b1,c1)). Despite the presence of surface voids, cross-sectional analysis (Figure 12c) confirms the film's adherence to the alumina substrate without detachment or oxidation fractures. This integrity is maintained even after 4 min (Figure 13a) and 10 min (Figure 13b) of ablation. After 30 min of ablation, a noticeable thinning of the cross-section (Figure 13c) is observed. During the ablation process, the possible oxidation reactions were shown as follows:

SiO_2_·B_2_O_3_(l)→SiO_2_(g) + B_2_O_3_(g)

### 3.4. Thin-Film Temperature Sensor Oxidation Resistance Ablation Test

**Figure 14:**
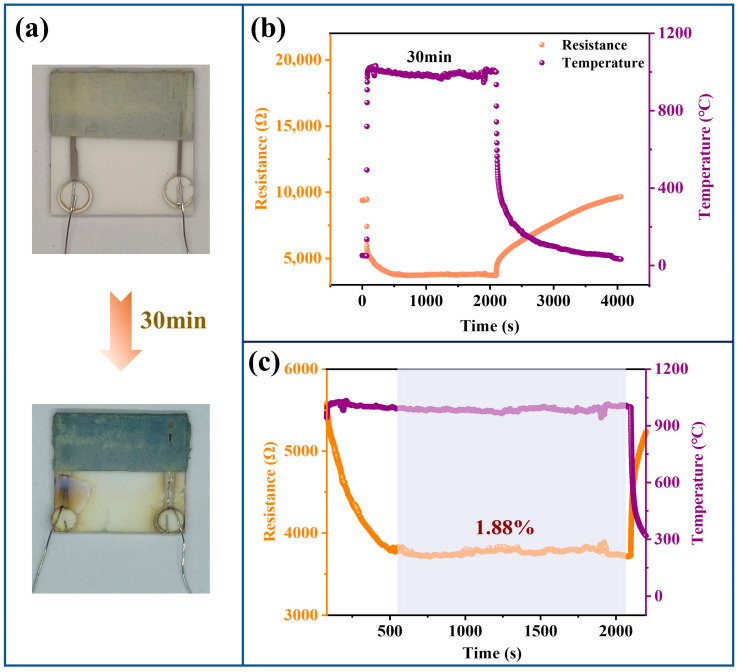
(**a**) Pre- and post-ablation film sensor; (**b**) Resistance variation of the film sensor during the ablation process; (**c**) Magnified view of (**b**).

**Figure 15:**
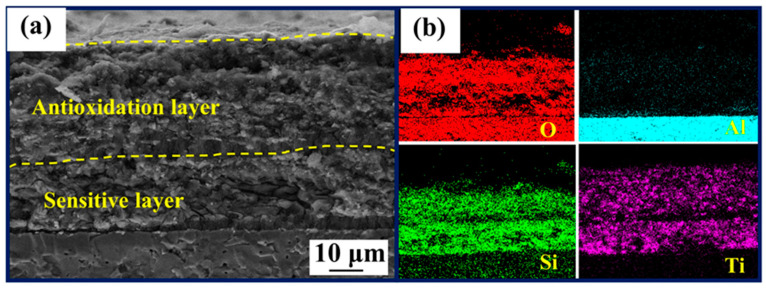
(**a**) Cross-sectional view of the film sensor after ablation; (**b**) EDS analysis of the cross-section.

By fabricating the thin-film coating directly on the alumina substrate, the oxidation and ablation exploration of the thin-film coating was completed. Finally, we used the electrical resistance characteristics of thin-film sensors to characterize the performance of ablation process coating. The thin-film coating was applied to the sensitive layer as an antioxidation layer forming a double-layer structure [16]. Figure 14a illustrates the optical image of the sensor prior to ablation, displaying a predominantly light yellow surface. After 30 min of ablation, the surface exhibited a deep blue color without any signs of cracking or delamination. Analysis of the film sensor's resistance variation demonstrated its impressive antioxidation performance even at the elevated temperature of 1000 °C (Figure 14b). Upon undergoing pyrolysis [15] at 1000 °C, the film sensor experienced a decrease in resistance, ultimately reaching a stable state with a rate of change of 1.88% over 25 min (Figure 14c). After the ablation process is completed, the resistance of the thin-film sensor can be restored to its initial value. The cross-sectional image after 30 min ablation of the sensor is shown in Figure 15a. The antioxidant layer remains firmly bonded to the sensitive layer without any cracks, despite the destruction of the protective glass layer formed on the surface of the film. In addition, the EDS analysis of the sensor cross-section (Figure 15b) shows a decrease in Si content above the antioxidant layer and the presence of a large amount of Si elements in the middle. Meanwhile, the Ti elements in the cross-section are uniformly distributed. During the ablation process, the vaporization of B_2_O_3_ decreased the surface temperature. Since SiO_2_ loses its stability above 2300 °C due to rapid evaporation [36,37], at 1000 °C, high viscosity SiO_2_ flows with the ablation gas stream and it is difficult to evaporate, and liquid SiO_2_ forms a dense layer on the ablation surface. By introducing Ti (TiB_2_), the film did not exfoliate significantly and the impact resistance of the film was enhanced.

**Figure 16:**
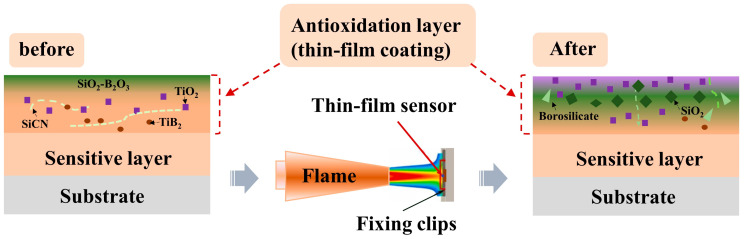
Schematic diagram illustrating the anti-ablation of the thin-film coatings.

To gain a comprehensive understanding of the oxidation and ablation resistance mechanism of the PDC/TiB_2_/B composite film, Figure 16 illustrates a simplified schematic. The thin-film coating is applied onto a sensitive layer that undergoes rapid oxidation and converts to Si, Ti, and B oxides at elevated temperatures. A highly viscous and fluid SiO_2_-B_2_O_3_ glass layer is swiftly formed, effectively blocking the infiltration of oxygen. During the ablation process, the film surface temperature rises rapidly, leading to the volatilization of B_2_O_3_, which results in the formation of ablation products, such as SiO_2_ and borosilicate, which are carried by the ablation airflow within and around the coating. While SiO_2_ exhibits low evaporation at 1000 °C, it remains deposited on the film surface, forming a high-viscosity SiO_2_ layer. This layer restricts the ingress of oxidizing gases into the film coating, thereby preventing further oxidation.

## 4. Conclusions

This study focuses on investigating the oxidation and ablation behavior of PDC/TiB_2_/B composites, which has an enhancing effect on the performance of PDC thin-film sensors. Through high-temperature heat treatment, a dense SiO_2_-B_2_O_3_ oxide layer forms on the surface of the thin-film coating, effectively preventing further oxidation of the sensitive layer by oxygen. After oxidation in air at 800 °C for 10 h, the sample experiences only a 0.2% mass loss. Moreover, the generated oxide layer is also critical to the improvement of the ablation resistance. During high-temperature ablation at 1000 °C, the B_2_O_3_ on the coating's surface absorbs and dissipates heat, leading to its significant volatilization. Simultaneously, the SiCN ceramic absorbs heat and undergoes further oxidation, resulting in the formation of new SiO_2_. This process replenishes the vaporized B_2_O_3_ and contributes to the enhanced ablation resistance of the coating. After being exposed to the butane flame for 15 min, the coating demonstrates remarkable resistance to ablation, with a linear ablation rate of 1.04 μm/min. And the thin-film sensor exhibits an impressive resistance change rate of 0.0752%/min at 1000 °C. Consequently, the particle-filled PDC composite film coating has a key role in improving the oxidation and ablation performance of thin-film sensors. This work also provides insights and guidance for the design and development of thin-film coatings in extreme environments with high application potential.

## Data Availability

The data presented in this study are available on request from the corresponding author.
